# Maternal and foetal outcomes among pregnant women hospitalised due to interpersonal violence: A population based study in Western Australia, 2002-2008

**DOI:** 10.1186/1471-2393-11-70

**Published:** 2011-10-12

**Authors:** Lynn B Meuleners, Andy H Lee, Patti A Janssen, Michelle L Fraser

**Affiliations:** 1 Curtin-Monash Accident Research Centre (C-MARC), School of Public Health, Curtin University, GPO Box U1987, Perth, Western Australia 6845, Australia; 2 School of Public Health, Curtin University, GPO Box U1987, Perth, Western Australia 6845, Australia; 3 Department of Health Care and Epidemiology, University of British Columbia, 2329 West Mall, Vancouver, British Columbia V6T 1Z4, Canada

## Abstract

**Background:**

Interpersonal violence is responsible for more ill-health and premature death in women under the age of 45 than other preventable health conditions, but findings concerning the effects of violence during pregnancy on both maternal and foetal health have been inconsistent.

**Methods:**

A retrospective population-based cohort study was undertaken using linked data from the Hospital Morbidity Data Collection and the Western Australian Midwives' Notification System from 2002 to 2008. The aim was to determine the association between exposure to interpersonal violence during pregnancy and adverse maternal and foetal health outcomes at the population level.

**Results:**

A total of 468 pregnant women were hospitalised for an incident of interpersonal violence during the study period, and 3,744 randomly selected pregnant women were included as the comparison group. The majority of violent events were perpetrated by the pregnant women's partner or spouse. Pregnant Indigenous women were over-represented accounting for 67% of all hospitalisations due to violence and their risk of experiencing adverse maternal outcomes was significantly increased compared to non-Indigenous women (adjusted odds ratio 1.53, 95% CI 1.21 to 1.95, p = 0.01). Pregnant women hospitalised for an incident of interpersonal violence sustained almost double the risk for adverse maternal complications than the non-exposed group (95% CI 1.34 to 2.18, p < 0.001). The overall risk for adverse foetal complications for pregnant women exposed to violence was increased two-fold (95% CI 1.50 to 2.76, p < 0.001).

**Conclusions:**

The risk of adverse health outcomes for both the mother and the baby increases if a pregnant woman is hospitalised for an incident of interpersonal violence during pregnancy.

## Background

Interpersonal violence is responsible for more ill-health and premature death in women under the age of 45 years than any other preventable health conditions such as hypertension, obesity and diabetes [1]. When interpersonal violence is experienced during pregnancy, it not only affects the health and well-being of the mother but is also associated with adverse health outcomes for the foetus [2]. This has significant ramifications because negative birth outcomes represent a significant cost to society. For example, low birth weight and pre-term infants contribute disproportionately to neonatal morbidity and health care costs, as well as leading to a multitude of short and long term health problems [3].

The term 'interpersonal violence' refers to "*the intentional use of physical force, or power, threatened or actual, against oneself, another person, or against a group or community, that either results in, or has a likelihood of resulting in injury, death, psychological harm, mal-development or deprivation*" [4]. This definition includes victimisation perpetrated against intimate partners, parents, siblings, children, other relatives, friends, acquaintances, colleagues and strangers [4-6]. Interpersonal violence during pregnancy may be perpetrated by current or previous intimate partners, family members, and strangers or may occur as a result of fighting. Previous research, however, has indicated that violence during pregnancy is more commonly perpetrated by an intimate partner [7-9].

The effects of violence during pregnancy on both maternal and foetal health have been extensively investigated in the literature. However, conflicting results have been reported, possibly due to limitations in sample sizes, study methodology and operational definitions [10,11]. Recent studies conducted in the USA used population-based data to examine the adverse effects of violence during pregnancy [12,13]. In Western Australia (WA), a population-based study reported a rising incidence of interpersonal violence hospitalisations particularly among women of child bearing age, highlighting this serious public health issue [14]. However, there has been minimal research specifically targeting this vulnerable group in Australia.

This population-based, retrospective cohort study utilised the Western Australian Data Linkage System to determine the association between exposure to interpersonal violence during pregnancy and adverse maternal and foetal health outcomes from 2002 to 2008.

## Methods

### Study design

A retrospective population-based cohort study was undertaken.

### Definition and databases

The study used administrative data from the Western Australian Data Linkage System which represents one of only a small number of record linkage systems in the world. It records longitudinal data on the use of health services and vital events for the entire Western Australian population of over 2.2 million people. De-identified data were obtained through the linkage of the Hospital Morbidity Data Collection (HMDC) and the Western Australian Midwives' Notification System. The HMDC contains information concerning all inpatient discharge summary data from all public and private hospitals in Western Australia from 1970 onwards. The Western Australian Midwives' Notification System contains the mother's demographic information, details of the pregnancy, labour, delivery, gestational age of baby and birth records (both live and death records) from 1980 onwards.

Cases consisted of all women who were pregnant and admitted to a WA hospital between January 2002 and December 2008 due to involvement in an incident of interpersonal violence. A case was identified as a 'victim of interpersonal violence' if the principal diagnosis for at least one hospital separation during pregnancy was an 'injury', as designated by a diagnosis code between S00.0 and T98.3 (Chapter XIX, ICD-10-AM), and a primary external cause indicating that at least one injury in the case record was inflicted by another person, as designated by external cause codes between X85 and Y09 (ICD-10-AM) [15]. Pregnancy was defined by ICD 10-AM codes 000-082, Z33, Z32.1, Z34-Z35, Z37. At least ten months of data before and after the hospital admission for violence were extracted from the hospital morbidity records. These records were then linked to the Western Australian Midwives' Notification System to identify maternal birth and foetal outcomes. The comparison group was randomly selected from women who had only been admitted to hospital for a pregnancy-related event including the delivery episode and had no diagnosis of interpersonal violence for that event or prior to or after the pregnancy. A look back period of 10 months before and after the delivery date was chosen to ensure there was no mis-classification. These records were then linked to the Western Australian Midwives' Notification System to identify maternal birth and foetal outcomes for that group.

In this study, adverse maternal birth outcomes included threatened abortion (<20 weeks), placental abruption, placental praevia, preterm labour, premature rupture of the membranes, and postpartum haemorrhage. Adverse foetal birth outcomes included foetal distress, low birth weight (less than 2500 g), infant death and foetal death.

The circumstance of the violence event was defined by the major injury grouping framework devised by the Center for Disease Control and Prevention. The external cause codes for injury inflicted by another was divided into four sub-groups designating the following methods of inflicting injury: 'by bodily force' (Y04 [ICD-10-AM]), 'by sharp or blunt object' (Y99, Y00 [ICD-10-AM]), 'by maltreatment or rape' (Y05, Y06.0-9, Y07.0-9 [ICD-10-AM]) and 'by other methods' (all other codes between X85 to Y09 (ICD-10-AM) [15]. Meanwhile, the relationship of the perpetrator to the victim was identified using the fifth digit classification of the external cause of injury codes.

This study was conducted in accordance to the guidelines of the Declaration of Helsinki. Ethical approval was obtained from both the Human Research Ethics Committee at Curtin University and the Data Linkage Branch of the Department of Health WA.

### Statistical analysis

Descriptive statistics were used to summarise the demographic profile of the sample, including circumstances of the injury event and perpetrator-victim relationship. Chi-squared tests were undertaken to compare demographic characteristics and maternal and foetal outcomes between pregnant women hospitalised for violence and those not hospitalised for violence. Odds ratios and confidence intervals were calculated using multivariable logistic regression models after accounting for potential confounders namely age, maternal smoking and Indigenous status which can affect maternal and foetal outcomes [10-13]. Two separate logistic regression models were undertaken. For the first model, the outcome of interest was adverse maternal outcomes which included threatened abortion (<20 weeks gestation), preterm labour (<37 weeks), pre-labour rupture of the membranes, postpartum haemorrhage (≥500 ml), placental previa, placental abruption, and other causes of antepartum haemorrhage. For the second model, the outcome of interest was adverse foetal outcomes which included foetal distress, infant death, low birth weight and foetal death. All statistical analyses were performed in the SAS package version 9.1 [16].

## Results

A total of 468 women were admitted to hospital after involvement in at least one incident of interpersonal violence while pregnant from 2002 to 2008. A comparison group of 3,744 pregnant women hospitalised for a pregnancy related event including delivery but who did not have a record for an incident of interpersonal violence throughout their pregnancy were randomly selected. The sample characteristics of both groups are presented in Table 1.

**Table 1:** Demographic, clinical and lifestyle characteristics of pregnant women hospitalised for interpersonal violence and a comparison group: Western Australia, 2002-2008

	Pregnant women hospitalised for interpersonal violence (n = 468)		Pregnant women not hospitalised for interpersonal violence (n = 3,744)		P****
	**n**	**%**	**n**	**%**	

**Women's age (years)**					<0.001
≤20	140	30.0	419	11.2	
21 - 25	148	31.6	685	18.3	
26 - 30	98	20.9	1,153	30.8	
31 - 35	50	10.7	1,017	27.2	
≥36	32	6.8	470	12.6	
**Indigenous status**					<0.001
Yes	311	66.5	193	5.2	
No	157	33.5	3,551	94.8	
Parity*					<0.001
0	88	18.8	1,637	43.7	
1	75	16.0	963	25.7	
2	82	17.5	558	14.9	
3+	223	47.7	586	15.7	
**Gestational age (weeks)**					<0.001
≤27	13	2.8	32	0.9	
28 - 32	18	3.9	44	1.2	
33 - 36	65	13.9	212	5.7	
≥37	372	79.5	3,456	92.3	
**Marital status**					<0.001
Yes**	183	40.9	2,823	76.3	
No***	264	59.1	879	23.7	
**Maternal smoking**					<0.001
Yes	276	59.0	691	18.5	
No	192	41.0	3,053	81.5	

* Parity is defined as the number of live-born children a woman has delivered; ** married or de facto relationship; *** widowed, divorced or single; **** P values from chi-squared tests

A large majority of pregnant women exposed to violence tended to be 25 years of age and under (61.6%), Indigenous (66.5%), multiparous (65.2%), smoked during their pregnancy (59.0%), and were not in a married or de-facto relationship (59.1%). The most common type of violence was inflicted by bodily force (65.8% of cases), followed by rape (15.4%), assault with a blunt/sharp object (11.1%) and other types of assault (11.1%). Of those cases where a code existed describing the relationship between the perpetrator and the victim (72.0%), the majority of pregnant women were assaulted by either their spouse or partner (69.5%), followed by a person where the relationship was not specified (20.8%), or another family member (5.6%) (not shown in the table).

Table 2 shows maternal and foetal outcomes by violence exposure status. Threatened abortions (<20 weeks) occurred significantly more often among women in the non-exposed group than the exposed group. Other maternal complications occurred significantly more often among women exposed to violence than non-exposed women during their pregnancy.

**Table 2:** Maternal and foetal outcomes among pregnant women hospitalised for interpersonal violence and a comparison group: Western Australia, 2002-2008

	Pregnant women hospitalised for interpersonal violence (n = 468)		Pregnant women not hospitalised for interpersonal violence (n = 3,744)		P***
**Outcomes**	**n**	**%**	**n**	**%**	

**Maternal**					
Threatened abortions (<20 weeks)	12	2.6	218	5.8	<0.001
Threatened preterm labour (<37 weeks)	42	9.0	96	2.6	<0.001
APH* - placenta praevia	5	1.1	28	0.7	0.46
APH - placental abruption	3	0.6	13	0.3	0.41
APH - other	16	3.4	110	2.9	0.56
Pre-labour rupture of membranes	43	9.2	210	5.6	<0.001
PPH** (≥500 ml)	68	14.5	356	9.5	<0.001
**Foetal**					
Foetal distress	65	13.9	599	16.0	<0.001
Low birth weight	115	24.6	243	6.5	<0.001
Foetal death	5	1.1	6	0.2	0.02
Infant death	1	0.2	26	0.7	0.35

* APH = Antepartum haemorrhage; ** PPH = Postpartum haemorrhage; *** P values from chi-squared tests

The non-exposed group of women reported a significantly higher percentage of foetal distress compared to women who had been exposed to violence during their pregnancy. Adverse foetal outcomes such as low birth weight and foetal deaths, however, occurred significantly more often among women exposed to violence during their pregnancy, compared to the non-exposed group.

### Risk of adverse maternal outcomes

As shown in Table 3, exposure to violence during pregnancy was associated with a 1.7 fold-increase in the risk of maternal complications (95% CI 1.34 to 2.18, p < 0.001). These complications included threatened abortions, preterm labour, antepartum haemorrhage (due to placenta praevia, placental abruption or other), pre-labour rupture of membranes and postpartum haemorrhage. The increase in risk was evident after accounting for potential confounders. Indigenous women also had a 1.5-fold increased risk of experiencing maternal complications relative to non-Indigenous women (95% CI 1.21 to 1.95, p = 0.01).

**Table 3:** Results of multivariable logistic regression for adverse maternal outcomes among pregnant women hospitalised due to interpersonal violence: Western Australia, 2002-2008

	Adverse maternal outcomes		
	**Adjusted odds ratio**	**P**	**95% CI**

**Exposed to violence**			
No*			
Yes	1.70	<0.001	1.34-2.18
**Maternal smoking**			
Yes*			
No	0.92	0.27	0.78-1.07
**Indigenous status**			
No*			
Yes	1.53	0.01	1.21-1.95
**Women's age (years)**			
≤20*			
21-25	1.06	0.62	0.85-1.32
26-30	1.04	0.70	0.84-1.29
31-35	1.12	0.32	0.90-1.39
≥36	1.26	0.08	0.98-1.62

* Reference group

### Risk of adverse foetal outcomes

As shown in Table 4, the risk of adverse foetal outcomes, which included low birth weight, foetal distress, and foetal/infant death, among women who had been hospitalised due to violence during their pregnancy, was double that of women who had not been exposed to violence (95% CI 1.50 to 2.76, p < 0.001). Similarly, Indigenous status was significantly associated with a 2-fold increased risk for adverse foetal outcomes. Non-smoking by the pregnant women significantly reduced the risk of experiencing negative foetal outcomes by 37% (95% CI 0.50 to 0.79, p < 0.001).

**Table 4:** Results of multivariable logistic regression for adverse foetal outcomes among pregnant women hospitalised due to interpersonal violence: Western Australia, 2002-2008

	Adverse foetal outcomes		
	**Adjusted odds ratio**	**P**	**95% CI**

**Exposed to violence**			
No*			
Yes	2.03	<0.001	1.50-2.76
**Maternal smoking**			
Yes*			
No	0.63	<0.001	0.50-0.79
**Indigenous status**			
No*			
Yes	2.04	<0.001	1.50-2.77
**Women's age (years)**			
≤20*			
21-25	1.02	0.89	0.74-1.41
26-30	0.92	0.61	0.67-1.26
31-35	1.25	0.17	0.91-1.73
≥36	1.25	0.25	0.85-1.82

* Reference group

## Discussion

The study has highlighted that pregnant women exposed to violence may sustain poor health outcomes for themselves and their baby. In this whole population study, pregnant women hospitalised for an incident of interpersonal violence were at almost double the risk of experiencing one or more adverse maternal complication than the non-exposed group. The findings provide further evidence of an association between antepartum hemorrhage and exposure to violence [17]. Violence is often directed towards the pregnant women's abdomen and the high prevalence of injury due to blunt force may explain these results [17] Consistent with previous research,[7-9] the majority of violent events were perpetrated by the pregnant women's partner or spouse. The overall risk of one or more adverse foetal complications among pregnant women exposed to violence was also increased 2-fold.

It is important to note the multifactorial relationship between violence and its impact on maternal and foetal outcomes during pregnancy.[9,12,13,18-27] Similar to the literature, [12,28-30] certain demographic characteristics were found to be associated with the risk of violence, such as younger age, marital status and parity. Population subgroups should be targeted for violence screening and counselling during pregnancy.

While mixed findings have been reported on the association between ethnicity and rates of interpersonal violence in the US and South America,[25,31,32] it is evident that significant differences exist between Indigenous and non-Indigenous pregnant women. Pregnant Indigenous women accounted for 67% of all hospitalisations due to violence despite representing approximately 4% of the WA population. This is consistent with previous research which identified that hospital admissions by Indigenous females due to violence were consistently higher than Indigenous males, non-Indigenous males and females [14]. High rates of established behavioural health factors such as being disadvantaged economically, broken family ties, smoking, alcohol and substance abuse increased the risk of experiencing interpersonal violence among Indigenous families,[33,34] which may explain the higher occurrence of interpersonal violence related hospitalisations among our cohort of Indigenous women. In addition, the risk of experiencing adverse maternal and foetal outcomes for this group was significantly increased. These findings have important implications for the planning of health services and distribution of resources to effectively reduce the burden of interpersonal violence in the Indigenous community.

This study has addressed a number of shortcomings of past research. The use of the WA Data Linkage System assisted in reducing selection bias, minimised loss to follow up and enabled the assessment of the association between pregnant women exposed to violence and adverse maternal and foetal outcomes at the population level. It also had the advantage of detecting small differences by inclusion of a large number of cases. Another strength was the use of high quality, objective data which should be more accurate than information obtained via participant self-report measures [35,36]. Moreover, the results obtained from this population-based study may be generalisable to the rest of Australia, because WA is considered to be representative of the broader Australian population in terms of key socio-demographic and health economic indicators [37].

Several limitations should be taken into account. Firstly, our findings reflect only the severe cases of interpersonal violence, namely those which led to hospitalisation. Therefore, findings cannot be generalised to pregnant women exposed to less severe violence not requiring hospitalisation. Also, many violent events in Australia are never reported,[38] particularly among Indigenous people and particularly incidents involving domestic violence. Limited access to hospitals in rural and remote areas may also result in underreporting [39]. We also acknowledge that women in the comparison group might have been exposed to violence during pregnancy that did not require hospitalisation or was not reported, consequently underestimating the risk of adverse outcomes associated with violence. Despite this, it is known that pregnant women are more likely to seek medical attention due to concern for their unborn baby [40]. This study investigated maternal and foetal outcomes for women who were victims of an acute episode of violence during pregnancy. Exposure to chronic violence, on the other hand, may affect pregnancy outcomes differently and warrant further studies.

Finally, a large number of factors other than interpersonal violence are known to contribute to poor maternal and foetal outcomes. These include alcohol and drug usage, living conditions, health conditions, nutrition, level of prenatal care and socioeconomic status. Adverse outcomes can occur as a result of interpersonal violence, biological, behavioural and socioeconomic factors [10]. However, information on such potential confounding factors was not captured in the available databases.

## Conclusions

In conclusion, pregnant women hospitalised for an incident of interpersonal violence are at significantly increased risk of adverse maternal and foetal outcomes. Since poor maternal and foetal outcomes can have long term health consequences, greater priority needs to be given to the primary prevention of violence against pregnant women and the care given to these women following an incident of violence. In developing a response to violence, the findings indicate that prevention programs should target younger pregnant women and focus on preventing intimate partner violence. It is imperative that culturally appropriate intervention programs are implemented to reduce the high rate of violence against Indigenous pregnant women and also that they receive appropriate care and monitoring after exposure to violence.

## Competing interests

The authors declare that they have no competing interests.

## Authors' contributions

LBM conceived of the study, participated in its design and co-ordination, acquired the data, analysed and interpreted data and was involved in the drafting and revising of the manuscript. AHL participated in the design of the study, in data analysis and revised the manuscript critically for intellectual content. PAJ participated in the study's conception and design, interpretation of data and revised the manuscript for intellectual content. MLF contributed to the design of the study, assisted in data acquisition and the drafting of the manuscript. All authors read and approved the final manuscript.

## Pre-publication history

The pre-publication history for this paper can be accessed here:

http://www.biomedcentral.com/1471-2393/11/70/prepub
